# Problematic social media use, everyday memory failures, and prospective and retrospective lapses: evidence from a large sample of young adults

**DOI:** 10.3389/fpsyt.2026.1839080

**Published:** 2026-06-12

**Authors:** Conchita Sisí, M. P. Fernández-Martín, Ståle Pallesen

**Affiliations:** 1 Department of Psychology, Faculty of Health Sciences-HM Hospitals, Camilo José Cela University, Madrid, Spain; 2 Department of Psychosocial Science, University of Bergen, Bergen, Norway; 3 Department of Psychology, Faculty of Health Sciences, UNIE University, Madrid, Spain; 4 Norwegian Competence Center for Gambling and Gaming Research, University of Bergen, Bergen, Norway

**Keywords:** cross sectional analysis, everyday memory failures, memory, PRMQ, problematic social media use (PSMU), retrospective memory, young adults

## Abstract

**Introduction:**

Problematic social media use (PSMU) has become a growing research topic due to its potential psychological and cognitive consequences. However, little research has examined its relationship with everyday memory functioning, particularly specific forms of memory.

**Methods:**

A sample of 943 Spanish young adults aged 18 -35 completed validated measures of PSMU, everyday memory failures, prospective memory lapses and retrospective memory lapses. Non-parametric analyses, group comparisons and mediation analyses with bootstrap resampling were conducted.

**Results:**

Higher PSMU was associated with more frequent everyday memory failures and with greater prospective and retrospective lapses. Everyday memory failures mediated a substantial proportion of the association between PSMU and both prospective and retrospective lapses. Participants meeting the proposed clinical cutoff for PSMU reported poorer memory functioning than those below this threshold.

**Discussion:**

These findings suggest that PSMU is associated with greater subjective memory difficulties in daily life, highlighting the relevance of everyday memory failures as a potential explanatory mechanism linking problematic social media use with prospective and retrospective memory problems.

## 1 Introduction

Over the past decade, social media use has expanded dramatically, with young adults emerging as the most engaged demographic group. In Spain, nearly 94% of individuals aged 18 to 24 report daily use of social media (1). While social media platforms support communication and creativity, excessive use has increasingly been associated with negative outcomes such as decreased well-being and addiction resembling behaviors (2–4). In this realm, problematic social media use (PSMU) refers to a pattern of excessive and difficult-to-control social media engagement that interferes with daily functioning (5). Though not officially classified as an addiction in modern psychiatric nosology, PSMU is often conceptualized through Griffiths’ (6) components model of behavioral addiction outlining six core criteria: salience, mood modification, tolerance, withdrawal, conflict, and relapse. Recent empirical studies have elaborated on this framework, linking PSMU to emotional dysregulation, sleep disturbances, and broader psychosocial impairments (7, 8). A comprehensive meta-analysis evidenced that PSMU is positively associated with anxiety, depression, and sleep problems, whereas general social media use is only weakly associated with mental health risks (7). However, despite increasing research on the emotional and behavioral implications of PSMU, its cognitive effects, particularly on memory, remain underexplored. The limited amount of previous research being conducted has shown that high levels of internet use are associated with impairments in domains such as decision-making, working memory, and inhibitory control (9, 10). Yet, many of these studies focus on problematic internet use overall, not PSMU specifically. Recent research suggests that excessive social media use may be associated with greater memory difficulties. Sharifian and Zahodne (11) found that on days when individuals spent more time on social media, they also reported more frequent everyday memory failures. Similarly, Tamir et al. (13) demonstrated that using social media during real-life experiences, such as taking and sharing photos, diminished later recall of the events. These findings suggest that engaging with social media may tax cognitive resources needed for memory encoding and retrieval processes. In particular, frequent multitasking and attentional switching while using social media may disrupt encoding processes, leading to weaker or more fragmented representations of ongoing experiences (e.g., difficulty recalling details of a conversation while simultaneously checking social media). In addition, retrieval processes may be affected, particularly in situations requiring spontaneous recall of recently encountered information (e.g., forgetting what one has just read or discussed). Over time, these disruptions may manifest as a broader pattern of everyday memory inefficiency. At the cognitive level, studies suggest that reliance on digital platforms may alter functional connectivity in memory-relevant brain regions. For instance, Ward et al. (12) reported that habitual dependence on online searches and social media reduced activity in areas associated with memory retrieval which aligns with the concept of “digital distraction,” in which constant alerts and fragmented attention may interfere with deep processing and information retention. Taken together, emerging evidence suggests that problematic social media behaviors could be associated with reductions in the quantity and clarity of retained information. Frequent interruptions, divided attention, and shallow encoding may contribute to everyday forgetfulness or memory impairment. However, little is known about how PSMU affects specific forms of memory functioning, such as retrospective (experiences with forgetting things that have happened) and prospective (experiences with forgetting to do things) lapses (14), both being essential for effective daily functioning. These gaps provide a strong rationale for further research, especially among young adults who are among the most intensive users of social media.

Specifically, PSMU is hypothesized to correlate positively with frequent everyday memory failures and with both retrospective and prospective lapses. Retrospective memory refers to the ability to recall past events or previously learned information (15). Prospective memory, by contrast, involves remembering to perform planned actions in the future, often referred to as “remembering to remember” (16). The latter includes forming an intention, retaining it over time, and executing it at the appropriate time (17). From a cognitive perspective, these disruptions may differentially affect distinct memory systems. For example, retrospective memory, which involves recalling previously encoded information or past experiences (often linked to episodic and declarative memory), may be impaired when encoding processes are weakened due to divided attention. In contrast, prospective memory, which relies on self-initiated retrieval processes and executive control, may be particularly vulnerable to frequent interruptions and habitual checking behaviors characteristic of problematic social media use (e.g., forgetting to carry out intended actions such as replying to messages or attending scheduled tasks). Both retrospective and prospective memory are crucial for managing academic, occupational, and social obligations, all being especially relevant during young adulthood. Although impairments in attention and everyday cognitive functioning have been associated with PSMU (9, 18, 19) relatively few studies have examined how PSMU relates differentially to distinct memory systems, particularly retrospective and prospective lapses. Most relevant research on digital behavior has primarily relied on broad indices of cognitive lapses or general attentional failures (11, 20), without assessing the impact on specific memory processes. This lack of specificity is especially pertinent given that retrospective and prospective memory depend on partially distinct cognitive mechanisms and are differentially sensitive to factors such as impulsivity, habitual checking behaviors, and impaired self-initiated control (21–23) Prior research has shown that prospective memory lapses are more frequent in individuals with high impulsivity or behavioral addiction profiles (18), which theoretically fit well with the behavioral characteristics of PSMU. However, the association between prospective lapses and PSMU has rarely been examined in research on PSMU. In parallel, everyday memory failures are conceptualized here as a broad class of self-reported cognitive slips in daily life, including failures of retrieval, task monitoring, conversational tracking, memory for activities, and other routine memory-related problems. These everyday memory failures may include concrete situations such as losing track of ongoing conversations, forgetting recently encoded information, or failing to remember routine tasks in daily life, all of which may be exacerbated by constant digital interruptions and attentional fragmentation. Taken together, these process-level distinctions provide a more precise framework for understanding how problematic social media use may be associated with both broad everyday memory failures and more specific prospective and retrospective memory lapses.

Thus, in the present study, everyday memory failures are treated as a broader and more heterogeneous construct than prospective and retrospective lapses, which refer more specifically to forgetting intended future actions and previously learned information, respectively. Importantly, this distinction also reflects differences in the underlying cognitive processes: everyday memory failures may capture disruptions in encoding and monitoring processes in daily contexts, whereas prospective and retrospective lapses more directly reflect failures in intention retrieval and recall of previously encoded information under conditions of attentional interference. This former and broader construct may be especially vulnerable to the constant distraction and attentional switching inherent in PSMU. Evidence suggests that individuals with higher levels of problematic or addictive social media use report more frequent everyday memory failures than those with lower levels (19), yet these findings are often interpreted in global terms, without clarifying whether such failures are linked to more specific prospective and retrospective memory lapses. Addressing this gap, the present study examined the association between PSMU and self-reported everyday memory failures, as well as prospective and retrospective memory lapses. Given the cross-sectional design, the present study focuses on examining associations between variables rather than inferring causal effects. In this context, prospective memory refers specifically to self-reported failures in executing intended future actions in daily life, whereas retrospective memory refers to failures in recalling previously encoded information or past events, as measured by the PRMQ. The following hypotheses were posited: H1: Higher levels of PSMU will be associated with greater everyday memory failures. H2: Higher levels of PSMU will be associated with a higher frequency of prospective and retrospective lapses. H3: Everyday memory failures will statistically mediate the association between PSMU and prospective and retrospective lapses. Specifically, higher levels of PSMU are expected to be associated with increased everyday memory failures, which in turn are expected to be associated with greater prospective and retrospective lapses, suggesting that broader everyday memory failures may statistically account for part of the association between PSMU and more specific memory difficulties (18). H4: Individuals who meet the clinical cut-off for PSMU will exhibit significantly higher levels of everyday memory failures and more frequent prospective and retrospective memory lapses compared to non-clinical users.

## 2 Methods

### 2.1 Participants

A sample of young adults aged 18 to 35 years from Spain was recruited for the study consisting of an online survey. An *a priori* power analysis was conducted using G*Power 3.1 (24). Assuming an alpha level of.05 (two-tailed), statistical power of.80, and a small effect size (f² = .02), a minimum sample size of 390 participants was required for a regression model with two predictors (PSMU and everyday memory failures) predicting memory lapses (prospective and retrospective). This estimation was used as an approximation for the primary mediation analyses conducted in the present study, in which PSMU predicted prospective and retrospective memory lapses through everyday memory failures. The remaining analyses (correlations and group comparisons) were considered complementary to the primary mediation model. A total of N = 943 participants completed the survey and were included in the analyses. Participants were recruited through the online platform *Prolific*, which was chosen due to its ability to implement pre-screening filters, ensuring that participants met the inclusion criteria. The platform also facilitates efficient payment processing and communication with participants. Eligibility criteria were set to ensure a targeted sample. Participants were required to have Spanish as their primary language and to hold a Spanish nationality. These criteria were necessary for the administration of the Spanish version of the BSMAS, which is validated for use in Spanish-speaking populations (25). Participants were excluded if they reported needing social media for professional purposes, had a diagnosed mental illness, or had a physical medical condition known to affect attention (two specific *ad hoc* questions regarding this matter - “*Do you use social media platforms (e.g., Instagram, TikTok, LinkedIn, X/Twitter, Facebook) as part of your professional duties or to manage your business activities?”, and “Have you ever been diagnosed by a healthcare professional with any mental or neurological condition that could affect attention or mood (e.g., ADHD, depression, anxiety, epilepsy, traumatic brain injury)?”).* This strengthened the validity of the attention measures and reduced potential confounding factors. All participants were required to have at least 10 prior submissions in *Prolific*, with a minimum of 90% approval rate to ensure they were familiar with the platform as well as to boost the quality of the submissions. For further analyses, participants were subdivided in two groups, differentiating between a clinical (BSMAS ≥ 24) and non-clinical group (BSMAS < 24) according to Luo and collaborator’s (26) criteria. Data collection was fully funded by the Vice-rectorate of Research at the Camilo José Cela University through a €5000 grant award. Data collection lasted for a total of 14 days, including a pilot testing phase. Participants had a mean age of 26.6 years (SD = 4.3), and age was normally distributed. Just above half (52.4%) of participants were male. The majority (76.6%) had university-level education. Daily social media use varied: the largest group (43.9%) reported 1–3 hours per day, with smaller proportions reporting <1 hour (16.7%) or >5 hours (11.5%). The mean score on the BSMAS was 15.91 (SD = 4.68). Only 5.2% of participants scored in the “clinical” range (BSMAS ≥ 24), hence the vast majority (94.8%) fell below this threshold.

### 2.2 Procedure

An initial pilot test was conducted with 25 participants *(Participants section) who* provided feedback on the testing and survey procedures, reporting on issues such as technical difficulties and ambiguities in the instructions. Based on their feedback, adjustments were made to the instructions to improve clarity, and the average completion time for the tasks was re-estimated. The updated survey- and test protocol was then used for the main study. Recruitment was completed within two weeks, including the pilot testing phase and re-adjustment following pilot testing. On average, participants completed the survey/testing in 19 minutes. Compensation rates adhered to *Prolific’s* guidelines to ensure ethical remuneration.

### 2.3 Instruments

#### 2.3.1 Socio-demographic data

Variables commonly linked to social media use were assessed (27, 28) including age, sex, highest completed educational level, marital status, social media use for work purposes, nationality, primary language, and daily social media use. Educational level was operationalized as the highest completed degree and categorized as: no formal education, mandatory/compulsory schooling, high school/Baccalaureate, university degree (Bachelor’s or Master’s), and Doctorate (PhD), respectively. Self-reported daily social media use was measured according to five categories: 0–29 minutes, 30 minutes–1 hour, 1.5–3 hours, 3.5–5 hours, or more than 5 hours per day. **Table 1** presents the socio-demographic characteristics of the sample.

**Table 1:** Socio-demographic characteristics of the sample.

		N	%
Sex	Men	494	52.4 %
	Women	449	47.6 %
Marital status	Married	79	8.4 %
	In a relationship	411	43.6 %
Currently Studying	Single	453	48.0 %
	Yes	452	47.9 %
	No	491	52.1 %
Educational Level	No Formal Education	5	0.5%
	Mandatory School	41	4.3%
	High School/Baccaulerate	116	12.3 %
	University Degree (Bachelors,Masters)	722	76.6%
	PhD	59	6.3%

#### 2.3.2 Bergen social media addiction scale (BMAS)

This scale was used to assess PSMU levels among participants. The BSMAS (29) consists of six items, each rated on a 5-point Likert scale, ranging from “very rarely” (1) to “very often” (5). It reflects core addiction criteria such as salience, mood modification, tolerance, withdrawal, conflict, and relapse. The scale has demonstrated adequate reliability and good validity in Spanish-speaking populations (25). For the current study, an α of.79 was obtained. Luo et al. (26) identified a score of 24 as the optimal cut-off to establish PSMU, which was used in the present study.

#### 2.3.3 Everyday memory questionnaire

Everyday memory failures were assessed using the 28-item version of the Everyday Memory Questionnaire, originally derived from the measure developed by Sunderland, Harris, and Baddeley (30). The EMQ is a self-report measure of broad memory-related failures in everyday life, capturing heterogeneous difficulties such as retrieval problems, failures in monitoring ongoing activities, conversational tracking problems, and lapses in everyday task performance. Previous work on the EMQ-28 has suggested a multidimensional structure, including factors such as retrieval, task monitoring, conversational monitoring, spatial memory, and memory for activities. In the present study, the composite score was used as a global index of subjective everyday memory failures, with higher scores indicating more frequent everyday memory failures. Items are rated on a 5-point scale ranging from 1 (*once or less in the last month*) to 5 (*once or more in a day*). The EMQ does not provide a clinical cut-off score; therefore, scores were analyzed continuously. In the present sample, internal consistency was high (Cronbach’s α = .89).

#### 2.3.4 The Prospective and retrospective memory questionnaire

It is a 16-item self-report instrument specifically designed to distinguish between prospective memory lapses (i.e., forgetting to carry out intended future actions) and retrospective memory lapses (i.e., forgetting previously learned information or past events) (31). In contrast to the EMQ, the PRMQ provides a more domain-specific assessment organized by the temporal orientation of the lapse. Eight items assess prospective lapses and eight assess retrospective lapses. In the present study, the Spanish adaptation validated by González-Ramírez and Mendoza-González (32) was used. Items are rated on a 5-point frequency scale, with higher scores indicating more frequent lapses. Subscale scores were computed by summing the corresponding items. Internal consistency in the present sample was good (Prospective α = .77; Retrospective α = .84; total α = .89).

### 2.4 Data collection and ethics

As indicated, participants completed the study through *Prolific*, with data collected via a secure online platform. All participants provided informed consent before commencing with the study. The study was approved by the institutional ethics review board of the Faculty of Health Sciences at the Camilo José Cela University, ensuring compliance with ethical guidelines for human research. Data collection was completed efficiently within a two-weeks’ timeframe, and participant feedback was consistently monitored to address any unforeseen issues promptly.

### 2.5 Statistical analysis

Descriptive statistics were calculated for all study variables, including BSMAS scores, everyday memory failures, prospective and retrospective lapses (PRMQ subscales), age, sex, daily social media use hours, and clinical cut-off groups. Preliminary analyses indicated that most continuous variables deviated from normality, with the exception of age. Given the distributional characteristics of the data, non-parametric statistical methods were employed to ensure a conservative analytical approach. Bivariate associations among the key continuous variables (BSMAS, EMQ, PRMQ Prospective, PRMQ Retrospective, age, and daily social media use hours) were assessed using Spearman’s rank-order correlation coefficients.

For group comparisons, Mann–Whitney U tests were used to compare two independent groups, and Kruskal–Wallis H tests were conducted for comparisons across multiple groups. Mann–Whitney U tests examined differences in BSMAS scores, EMQ subscales, and PRMQ subscales between participants scoring below versus at/above the clinical cut-off for PSMU (BSMAS ≥ 24). Kruskal–Wallis tests were used to evaluate differences in these variables across the five categories of daily social media usage duration (ranging from less than 30 minutes to more than 5 hours per day). To examine the proposed associations in line with H3, mediation analyses were conducted to test whether a broader index of everyday memory failures accounts for part of the association between PSMU and more specific prospective and retrospective memory lapses. To assess potential multicollinearity between EMQ and PRMQ variables, variance inflation factors (VIF) were calculated. Given the cross-sectional design of the study, these mediation analyses were interpreted in terms of statistical associations rather than causal mechanisms. Two separate mediation models were estimated, with PRMQ Prospective and PRMQ Retrospective scores serving as dependent variables, respectively. In each model, BSMAS scores were specified as the independent variable (X), the total EMQ score was entered as the mediator (M), and the respective PRMQ subscale served as the dependent variable (Y). Indirect effects were estimated using a bootstrap resampling procedure with 5,000 samples to generate 95% confidence intervals (CI). Bootstrapping was used because it does not assume normality of the sampling distribution of the indirect effect, making it appropriate when variables deviate from normality. Mediation was considered statistically significant if the confidence interval for the indirect effect did not include zero. The proportion mediated was calculated as the ratio of the indirect effect to the total effect (ab/c) and expressed as a percentage. All statistical tests were two-tailed with a significance level set to *p* <.05. Analyses were performed using IBM SPSS Statistics (Version 25.0).

## 3 Results

### 3.1 Descriptive analyses

Descriptive analyses were performed to calculate the mean, standard deviation and distribution of BSMAS scores, sex, daily hours of social media usage, EMQ, PRMQ (Prospective and Retrospective) as well as educational level.

Descriptive statistics for the main study variables are presented in **Table 2**. Mean BSMAS scores were slightly higher among females than males, whereas EMQ and PRMQ scores were broadly similar across sex groups. Across categories of self-reported daily social media use, mean BSMAS, EMQ, and PRMQ scores tended to be higher in the groups reporting greater use, with the highest mean values generally observed among participants reporting more than 5 hours per day. Similarly, participants in the clinical BSMAS group (≥ 24) showed higher mean EMQ and PRMQ scores than those in the non-clinical group. Age was similar across groups and categories. Spearman’s correlations were calculated to evaluate associations among BSMAS scores, everyday memory failures and prospective and retrospective lapses as well as age and daily social media use. To assess potential multicollinearity between the variables, variance inflation factors (VIF) were calculated. VIF values were low (all VIFs < 2.5), indicating no problematic multicollinearity between the variables.

**Table 2:** Descriptive results for the main variables: (sex, educational level, daily social media usage in hours and PSMU).

*Variable*	*Category*	*BSMAS Mean (SD)*	*EMQ Mean (SD)*	*Prospective PRMQ Mean (SD)*	*Retrospective PRMQ Mean (SD)*	*Age Mean (SD)*
Sex	Male (n = 494)	15.1 (4.7)	14.2 (8.3)	20.4 (5.2)	18.4 (5.0)	26.5 (4.4)
	Female (n = 449)	16.8 (4.5)	14.7 (8.5)	21.7 (5.3)	19.3 (5.3)	26.7 (4.2)
Daily social media use (self-reported hours/day)	0–30 min (n = 34)	10.0 (3.1)	9.5 (7.1)	20.3 (5.3)	17.8 (5.6)	27.9 (4.1)
	30 min–1 hr(n = 124)	13.0 (4.0)	12.1 (7.6)	19.9 (4.6)	17.7 (4.1)	27.4 (4.6)
	1–3 hrs (n = 414)	15.6 (4.2)	13.9 (8.1)	20.7 (4.9)	18.5 (4.6)	26.3 (4.3)
	3–5 hrs (n = 263)	17.4 (4.4)	15.3 (8.4)	21.4 (5.2)	19.3 (5.5)	26.2 (4.2)
	> 5 hrs (n = 108)	18.7 (4.8)	17.6 (9.2)	22.8 (6.7)	20.4 (6.8)	25.8 (4.0)
PSMU Group (BSMAS cut-off)	Non-Clinical (n = 894)	15.4 (4.2)	13.8 (8.0)	20.8 (5.1)	18.6 (5.0)	26.6 (4.3)
	Clinical (n = 49)	25.4 (1.4)	21.5 (9.7)	24.4 (6.4)	22.4 (6.6)	26.0 (4.5)

*BSMAS, Bergen Social Media Addiction Scale (range: 6–30); higher scores indicate greater problematic social media use.* EMQ, Everyday Memory Questionnaire (EMQ-28 total). PRMQ, Prospective and Retrospective Memory Questionnaire; PRMQ Prospective and PRMQ Retrospective refer to the 8-item subscales*. “Daily Social Media Use” was assessed in five ordinal categories. The clinical group is defined as BSMAS ≥ 24. .*

### 3.2 Correlation analyses

BSMAS scores were positively correlated with everyday memory failures (ρ = .40, p <.001) and prospective and retrospective lapses (ρ = .35, p <.001). Everyday memory failures were strongly correlated with both prospective lapses (ρ = .67, p <.001) and retrospective lapses (ρ = .68, p <.001). Prospective and retrospective lapses were also highly correlated with each other (ρ = .75, p <.001). Daily social media use was also positively correlated with BSMAS scores (ρ = .40, p <.001) and showed small positive correlations with everyday memory failures and prospective lapses (ρ = .22 and.24), and a weaker, still positive, association with retrospective lapses (ρ = .11). Age was only weakly related to the main study variables.

### 3.3 Mediation analysis

Two mediation analyses were conducted using PROCESS Model 4 (33) to test Hypothesis 3. BSMAS was specified as the predictor (X), everyday memory failures (EMQ) as the mediator (M), and PRMQ prospective and retrospective lapses as outcomes (Y), respectively, in two separate models. Indirect effects were estimated using 5,000 bootstrap samples with 95% confidence intervals. Bootstrapping procedures were used as they do not assume normality of the sampling distribution of the indirect effect and are recommended when variables deviate from normality.

#### 3.3.1 Model 1: prospective memory lapses

**Figure 1:**
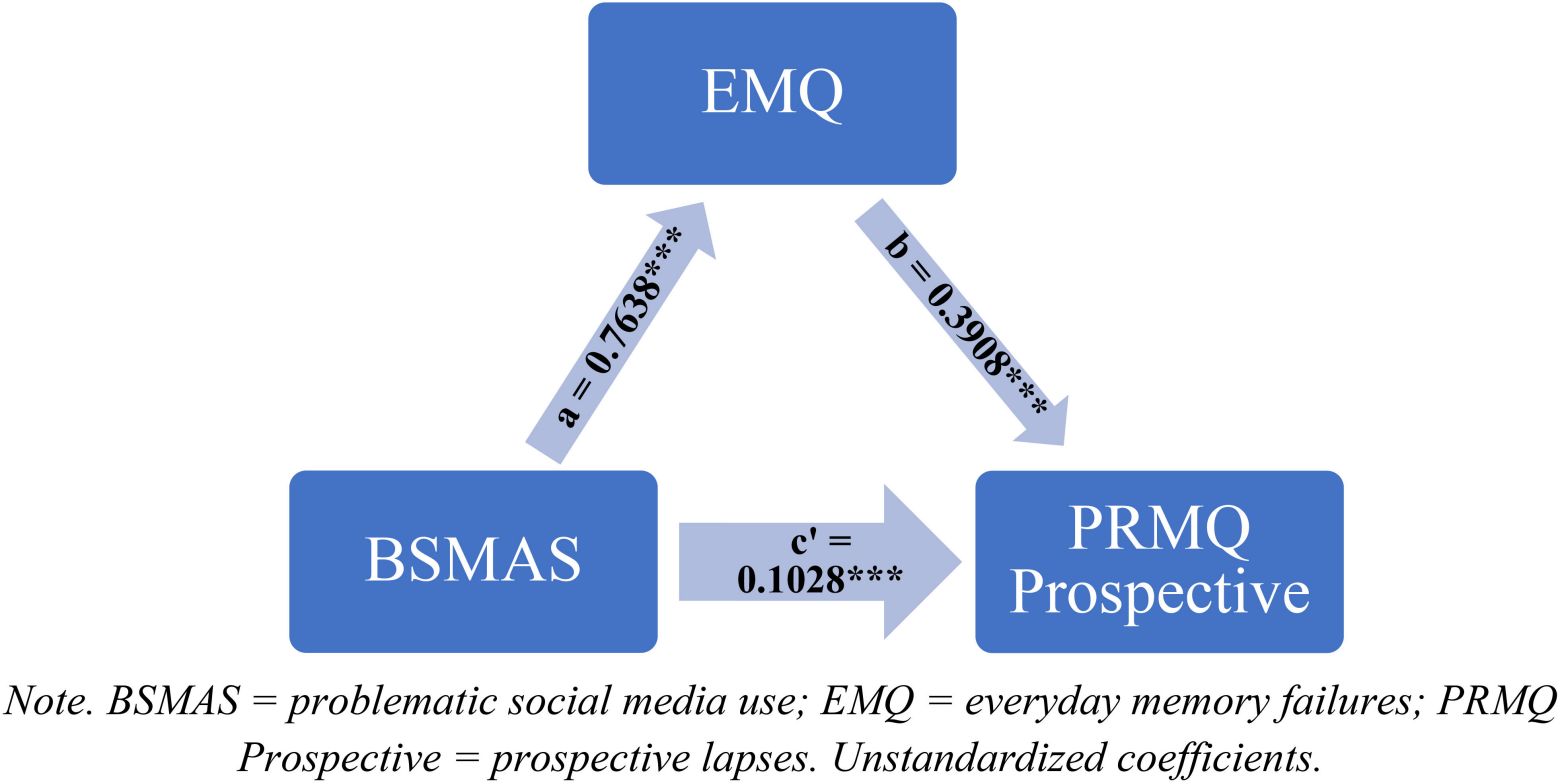
Mediation model for prospective memory lapses. BSMAS, problematic social media use; EMQ, everyday memory failures; PRMQ Prospective, prospective lapses. Unstandardized coefficients.

BSMAS scores were significantly associated with everyday memory failures (a = 0.7638, SE = 0.0562, t = 13.58, p <.001). Everyday memory failures was significantly associated with PRMQ prospective scores controlling for BSMAS (b = 0.3908, SE = 0.0152, t = 25.73, p <.001). The total effect of BSMAS on prospective lapses was significant (c = 0.4013, SE = 0.0342, 95% CI [0.3343, 0.4683]). The direct effect remained significant but reduced (c′ = 0.1028, SE = 0.0287, 95% CI [0.0466, 0.1590]), indicating partial mediation. The indirect effect was significant (ab = 0.2985, SE = 0.0268, 95% CI [0.2465, 0.3519]). The proportion mediated (ab/c) was 74.4%, indicating that nearly three quarters of the association between PSMU and prospective lapses was explained by everyday memory failures. The mediation model is presented in **Figure 1**.

#### 3.3.2 Model 2: retrospective memory lapses

**Figure 2:**
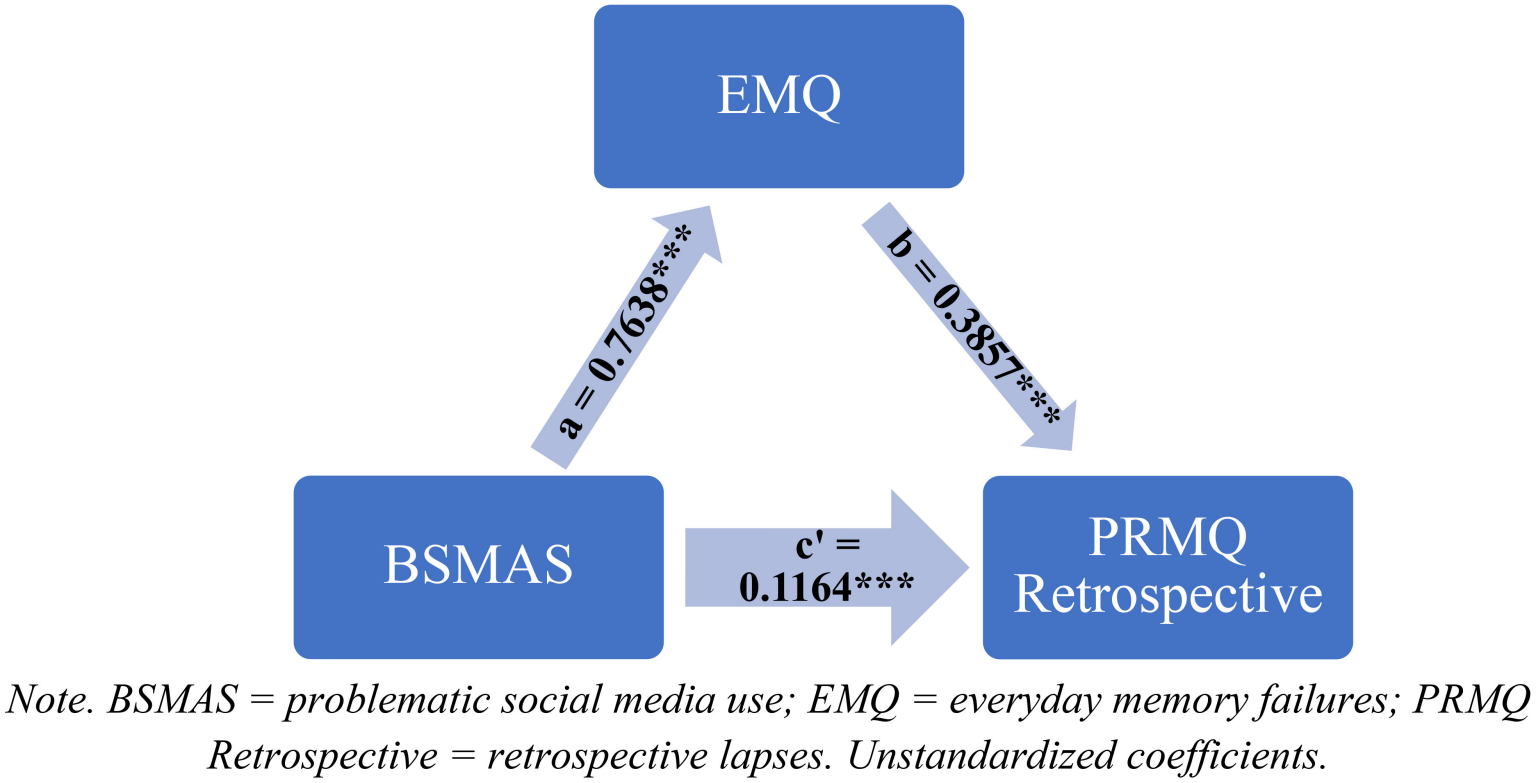
Mediation model for retrospective memory. BSMAS, problematic social media use; EMQ, everyday memory failures; PRMQ Retrospective, retrospective lapses. Unstandardized coefficients.

The same pattern emerged for retrospective lapses. BSMAS scores were significantly associated with everyday memory failures (a = 0.7638, SE = 0.0562, p <.001), which in turn were associated with PRMQ retrospective scores controlling for BSMAS (b = 0.3857, SE = 0.0154, p <.001). The total effect was significant (c = 0.4110, SE = 0.0345, 95% CI [0.3432, 0.4788]). The direct effect remained significant but smaller (c′ = 0.1164, SE = 0.0291, 95% CI [0.0593, 0.1735]). The indirect effect was significant (ab = 0.2946, SE = 0.0265, 95% CI [0.2439, 0.3501]). The proportion mediated was 71.7%, indicating substantial partial mediation. The mediation model for retrospective memory lapses is presented in **Figure 2**.

### 3.4 Clinical vs. non-clinical BSMAS groups

**Figure 3:**
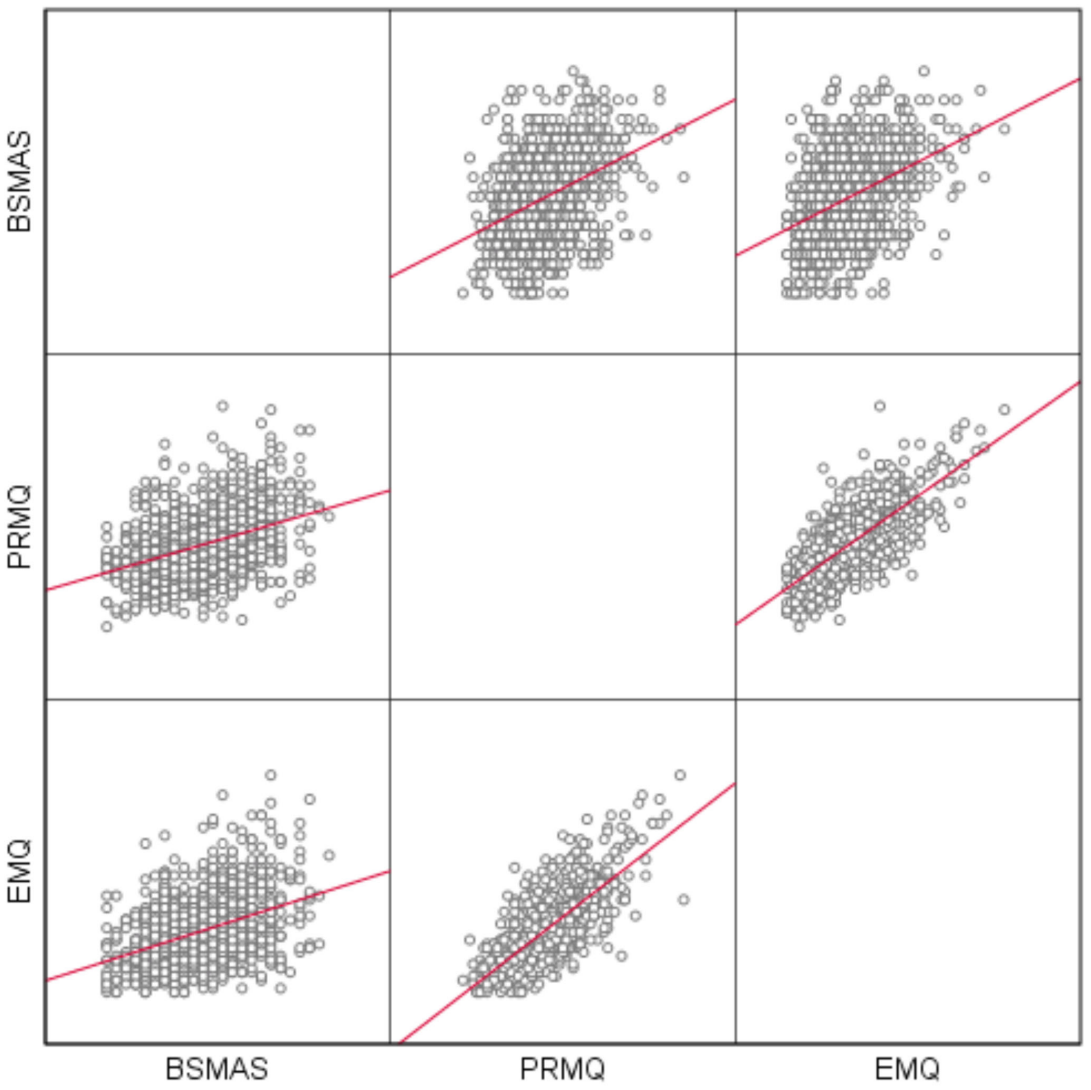
Associations among problematic social media use (BSMAS), prospective/retrospective lapses (PRMQ), and everyday memory failures (EMQ).

Participants scoring at or above the clinical cutoff (26) for PSMU (BSMAS ≥ 24) reported significantly higher levels of everyday memory failures and prospective and retrospective lapses compared to non-clinical users. Specifically, participants with clinically elevated scores reported higher levels of everyday memory failures (EMQ; U = 14,315, p <.001), higher scores on prospective lapses (PRMQ Prospective; U = 14,202, p <.001), and higher scores on retrospective lapses (PRMQ Retrospective; U = 14,714, p <.001). These comparisons are presented in **Table 3** and suggest that individuals with clinically elevated PSMU experience more frequent subjective memory difficulties across both prospective and retrospective domains. The associations among BSMAS, EMQ, and PRMQ variables are illustrated in **Figure 3**.

**Table 3:** Comparison of everyday memory failures (EMQ) and prospective and retrospective memory lapses (PRMQ) between clinical and non-clinical problematic social media users.

Outcome	Non-clinical Mdn	Clinical Mdn	U	p	Effect size (*r*_rb_)
EMQ	13.0	22.0	14,315	< .001	.35
PRMQ Prospective	21.0	24.0	14,202	< .001	.35
PRMQ Retrospective	18.0	22.0	14,714	< .001	.33

### 3.5 Differences across daily social media use categories

**Figure 4:**
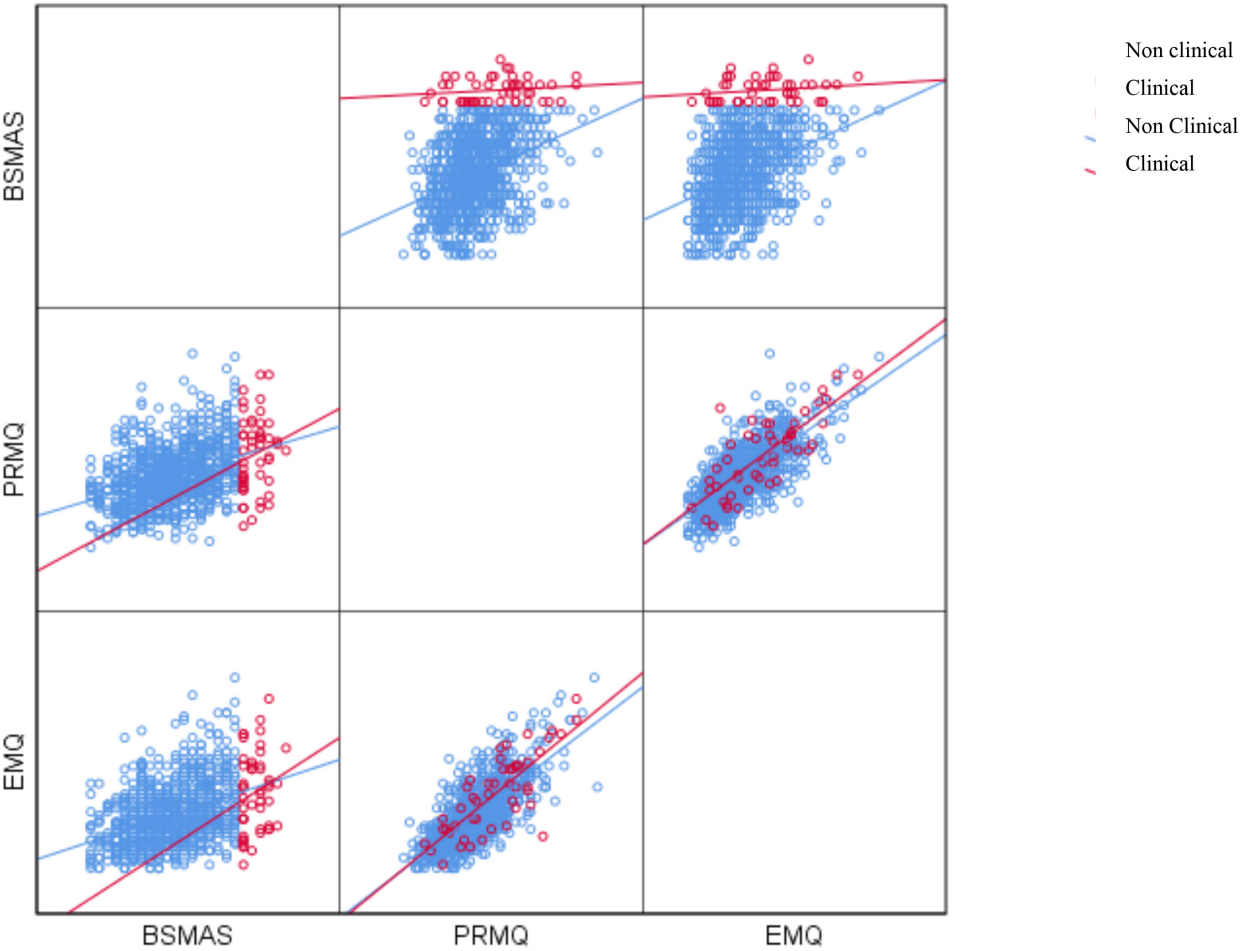
Associations among PSMU, prospective/retrospective lapses, and everyday memory failures.

Kruskal–Wallis tests revealed significant differences across daily social media use categories for all outcome variables. A strong association was observed for the BSMAS scores, χ²(4) = 157.80, p <.001, with participants reporting more than 5 hours per day exhibiting the highest BSMAS scores and those using less than 30 minutes per day the lowest. A similar graded pattern was found for everyday memory failures, χ²(4) = 18.50, p = .001, as well as for both prospective and retrospective lapses (PRMQ Prospective and PRMQ Retrospective; both p’s <.001). In particular, individuals reporting more than 5 hours social media use per day showed significantly higher levels of memory (everyday failures and retrospective and prospective lapses) compared to those using less than 1 hour per day (pairwise *post hoc* comparisons, adjusted p’s <.01). These results suggest a graded association between time spent on social media and subjective memory difficulties. **Figure 4** illustrates the associations among the variables separately for the clinical and non-clinical groups. The results of the Kruskal–Wallis analyses are presented in **Table 4**.

**Table 4:** Kruskal–Wallis tests across daily social media use categories.

Outcome	χ²	df	p	Effect size (ϵ²)	Post hoc summary
BSMAS	157.80	4	< .001	.164	> 5 h > all other groups
EMQ	18.50	4	.001	.015	> 5 h > < 1 h
Total PRMQ	21.90	4	< .001	.019	> 5 h > < 1 h
PRMQ Prospective	17.62	4	< .01	.015	> 5 h > < 1 h
PRMQ Retrospective	10.61	4	< .001	.007	> 5 h > < 1 h

## 4 Discussion

This study examined the links between PSMU and self-reported memory functioning in young adults, focusing on everyday memory failures and prospective/retrospective lapses. The findings provided support for all four hypotheses and converged on a main conclusion: PSMU was associated with more frequent everyday memory failures as well as prospective and retrospective lapses. Regarding H1, we predicted that higher PSMU would be associated with greater everyday memory failures (EMQ). This hypothesis was supported. Individuals with higher PSMU scores reported significantly more frequent everyday failures. The association was moderate (Spearman ρ = .4) and statistically significant. H2 proposed that higher PSMU would also be positively related to prospective and retrospective lapses which was also supported by the data. Participants reporting more problematic engagement with social media reported more prospective lapses (to remember intended future actions) and more retrospective lapses (to recall previously encountered information). These associations were comparable in magnitude to those observed for everyday memory failures, suggesting that the cognitive correlates of PSMU extend across multiple memory domains. H3 examined whether everyday memory failures would mediate the relationship between PSMU and prospective/retrospective lapses. The mediation analyses provided clear support for this hypothesis. Everyday memory failures substantially mediated the relationship between PSMU and both prospective and retrospective lapses. Approximately three-quarters of the association between PSMU and prospective lapses (74.4%) and nearly three-quarters of the association with retrospective lapses (71.7%) were explained by broader everyday memory failures. This pattern suggests that PSMU can be associated with poorer general day-to-day cognitive functioning, which is also reflected in more specific lapses related to remembering intentions and recalling recent information. In this sense, everyday memory failures may help explain the association between problematic digital behavior and more specific memory difficulties. Still, it is also important to have in mind the conceptual relationship between the two memory measures used in this study. As shown in **Table 5**, the EMQ-28 total score was highly associated with both PRMQ subscales, indicating that individuals who report more everyday memory failures also tend to report more prospective and retrospective lapses. This pattern is not unexpected, as both instruments assess subjective memory difficulties in everyday life. However, the two measures differ in scope. The EMQ-28 captures a broader range of everyday memory failures, including difficulties such as retrieval problems, failures in monitoring ongoing tasks or conversations, and memory for routine activities, whereas the PRMQ specifically distinguishes between prospective lapses (forgetting to carry out intended actions) and retrospective lapses (forgetting previously encountered information). In this sense, the EMQ-28 can be understood as reflecting a more general pattern of everyday memory inefficiency, whereas the PRMQ captures more specific forms in terms of retrospective and prospective memory lapses organized by the temporal orientation of the lapse. The high correlations observed between these measures therefore likely reflect their conceptual relatedness rather than complete redundancy/overlap. Taken together, these findings support the view that EMQ and PRMQ capture related but conceptually distinct aspects of subjective memory functioning, rather than reflecting redundant measures of the same construct. Finally, H4 addressed differences between clinical and subclinical levels of PSMU. Guided by recent proposals for clinical classification (e.g., 26), we expected individuals meeting a suggested clinical cutoff to show poorer memory outcomes. Data lent support for this hypothesis. Participants at or above the proposed clinical threshold displayed substantially poorer memory functioning, nearly a full standard deviation lower than those scoring in the non-clinical range. To our knowledge, this is the first empirical report documenting such a pronounced cognitive gap in memory functioning between clinically defined PSMU users and those below the threshold. This finding suggests that once social media use escalates to clinically relevant levels, cognitive consequences may be reflected in increased subjective memory difficulties, specifically retrospective and prospective lapses and everyday memory failures. This pattern may have implications for clinical practice. If individuals with clinically elevated PSMU consistently report substantial subjective memory difficulties, these complaints could serve as a useful behavioral indicator of PSMU severity. In applied settings, the assessment of everyday memory failures may help identify individuals at risk of problematic social media use, particularly when self-reported usage is unreliable or minimized. Moreover, memory complaints are often more tangible and salient for individuals than abstract constructs such as “problematic use,” which may facilitate clinical engagement. From an intervention perspective, these findings also suggest that targeting attentional control and everyday cognitive functioning could be a relevant component of approaches aimed at reducing PSMU. However, it remains unclear whether these difficulties reflect objective cognitive impairment, subjective perception biases, or both, highlighting the need for future research combining self-report and performance-based measures. Importantly, all key variables in the present study were assessed via self-report, which raises the possibility of shared method variance. It cannot be ruled out that the observed associations partly reflect a general tendency toward negative self-evaluation or response bias (e.g., greater self-criticism), rather than differences in actual memory performance. Beyond these clinical implications, the present findings also contribute to the broader theoretical understanding of how PSMU relates to cognitive functioning. Although concerns about digital media overuse and cognition are not new, relatively few studies have examined PSMU specifically, in contrast to general smartphone or internet use, constructs that are conceptually and clinically distinct (34). Moreover, memory has often been considered in broad cognitive terms, with limited attention to prospective and retrospective lapses in the context of social media. By simultaneously evaluating everyday memory failures together with more specific prospective and retrospective lapses within a mediation framework, the present study extends previous research by showing that everyday memory failures may help explain how PSMU is associated with more specific memory lapses. Our findings align with prior research showing that heavier digital media use is associated with greater subjective memory difficulties. For example, daily-diary evidence from Sharifian and Zahodne (11) showed more everyday memory failures on high social-networking days, while Farchakh et al. (35) reported similar associations between PSMU and self-rated memory difficulties in a Lebanese sample, suggesting this reflects a cross-cultural phenomenon. Experimental work further supports these associations: Dagher et al. (2021) found that media engagement during real-world events was associated with reduced memory encoding, potentially due to attentional diversion. Similarly, Reed (19) reported that higher scores on social media addiction measures were positively associated with frequent prospective and retrospective memory lapses, as well as everyday memory failures. The present results from the mediation analyses extend this literature by suggesting that broader everyday memory failures may represent one way of understanding the association between PSMU and more specific memory complaints. These everyday memory failures refer to a heterogeneous range of self-reported cognitive slips in daily life, including difficulties such as retrieval problems, failures in monitoring ongoing tasks, conversational tracking difficulties, impaired memory for routine activities, and other everyday memory-related problems. This broader pattern of subjective memory inefficiency may help explain why higher PSMU is associated with more specific prospective and retrospective lapses. Several processes may help explain these associations. Continuous alerts and task-switching implied in social media platforms may disrupt sustained attention, which is critical for encoding and retrieval processes. Repeated attentional diversion may lead to fragmented encoding and weaker consolidation of information, contributing to a more generalized experience of memory inefficiency in daily life. Such broader everyday memory problems may be reflected in specific difficulties remembering to carry out intended actions (prospective lapses) or recalling previously encountered information (retrospective lapses). Heavy digital engagement may also promote superficial processing or cognitive offloading, increasing reliance on external devices rather than internal memory systems. Neuroimaging findings (e.g., Liu et al., 2018) suggest altered connectivity in memory-related networks among heavy users, potentially weakening consolidation processes. Emotional and physiological factors, including stress, anxiety, or disrupted sleep, may further exacerbate these difficulties, although such variables were not directly measured in the present study. Taken together, the current findings suggest that a broad pattern of everyday memory failures may help explain the association between PSMU and more specific prospective and retrospective memory lapses.

**Table 5:** Spearman correlations among BSMAS, memory variables, age, and daily social media use.

Variable	BSMAS	EMQ	PRMQ Prospective	PRMQ Retrospective	Age	Daily social media use (hours)
BSMAS	—					
EMQ	0.40***	—				
PRMQ Prospective	0.35***	0.67***	—			
PRMQ Retrospective	0.35***	0.68***	0.75***	—		
Age	0.02	-0.05	-0.08*	-0.07*	—	
Daily social media use (hours)	0.40***	0.22***	0.24***	0.11*	-0.03	—

*BSMAS, Bergen Social Media Addiction Scale (range: 6–30); higher scores indicate greater problematic social media use.* EMQ, Everyday Memory Questionnaire (EMQ-28 total). PRMQ, Prospective and Retrospective Memory Questionnaire; PRMQ Prospective and PRMQ Retrospective refer to the 8-item subscales. “Daily Social Media Use” was assessed in five ordinal categories ordinal self-reported hours of social media per day (1 = 0–30 min, 5 = > 5 h). Note. ***p < .001. * p< 0.05.

## 5 Conclusion

In summary, the present study shows that PSMU can be associated with subjective memory difficulties in daily life, including both a broad range of everyday memory failures and more specific prospective and retrospective lapses. In the present study, these broader everyday memory failures refer to self-reported cognitive slips such as difficulties retrieving recently encountered information, problems monitoring ongoing tasks or conversations, and failures in remembering routine daily activities. Our findings indicate that a substantial portion of the association between PSMU and more specific memory lapses is statistically accounted for by this broader pattern of everyday memory-related difficulties. In other words, PSMU is positively associated with a more generalized experience of everyday memory inefficiency, which in turn is linked to more specific difficulties remembering to carry out intended actions (prospective lapses) and recalling previously encountered information (retrospective lapses). These findings are consistent with the idea that problematic social media use may be associated with disruptions in underlying cognitive processes such as encoding, retrieval, and attentional control, which may contribute to both generalized and more specific forms of subjective memory difficulties.

## 6 Strengths and limitations

This study offers several strengths. It is among the first empirical investigations to examine PSMU, not general smartphone or internet use, and its associations with prospective and retrospective lapses, as well as everyday memory failures using validated instruments in a relatively large (N = 943) sample. The simultaneous assessment of different memory components, coupled with the application of a recently proposed clinical cutoff for PSMU (26), allowed us to explore cognitive functioning across PSMU severity levels. Importantly, by modeling everyday memory failures as a mediator between PSMU and specific memory lapses, the study provides an initial framework for understanding potential cognitive pathways underlying PSMU. To our knowledge, no prior study has simultaneously assessed PSMU using psychometrically validated measures and examined its associations with both general memory failure and domain-specific memory lapses within a mediation framework, positioning this work as an important contribution to the emerging literature. Despite these strengths, a few limitations deserve mention. Due to the cross-sectional design, causal inferences cannot be drawn; it remains unclear whether PSMU contributes to memory failures, whether memory lapses increase vulnerability to problematic use, or whether a bidirectional relationship may be at play or whether the relationships may be better explained by various third variables. Additionally, although the cognitive measures included in the study were appropriate for detecting memory-related difficulties, they were relatively brief, which may limit the sensitivity of the results to subtle deficits. All data were based on self-report measures, which are vulnerable to various biases, including common method bias (36), recall bias (37), and social desirability bias (38). In addition, the use of a convenience sample places some limitations on the generalizability of the findings. The sample was relatively highly educated, with most participants (over 76%) holding a university degree. As such, the present findings may not fully generalize to young adults with lower educational attainment or to individuals with less developed self-monitoring or introspective abilities. Future research should aim to replicate these findings in more diverse and representative samples to assess their broader applicability. Furthermore, the categorization of daily social media use into predefined time ranges resulted in small gaps between categories (e.g., 61–89 minutes and 181–209 minutes). Consequently, some usage durations were not explicitly represented, which may have slightly reduced the precision in capturing participants’ exact time of use. Finally, the study did not control for potentially relevant confounders, such as sleep duration and quality, emotional problems, or stress, which have been independently linked to both PSMU and cognitive functioning. In addition, although participants with diagnosed mental health conditions were excluded, it is likely that individuals with subclinical or undiagnosed symptoms of anxiety or depression were still included. Given that these factors are associated with subjective memory complaints, their presence may have introduced additional uncontrolled variance in the observed associations. Within the scope of the present study, the analyses focused on bivariate and mediation models without incorporating additional control variables such as depression, anxiety, or sleep. Future work should adopt multivariate approaches to examine the unique contribution of PSMU while accounting for these potential confounding factors. In addition, longitudinal or experimental designs and more extensive cognitive protocols would help refine the causal mechanisms underlying these associations. More fine-grained and specific memory assessments in future surveys and experiments may further clarify the particular memory processes affected by PSMU. A further limitation concerns the use of self-report measures of everyday memory functioning. Although the EMQ-28 was conceptualized in this study as a broader index of everyday memory failures and the PRMQ as a more domain-specific measure of prospective and retrospective lapses, both instruments rely on subjective reports of cognitive difficulties in daily life. Future research could complement these measures with objective cognitive tasks reflecting these memory functions to further clarify the mechanisms underlying the observed associations.

## Data Availability

The raw data supporting the conclusions of this article will be made available by the authors, without undue reservation.
